# Guidelines for Physical Activity—A Cross-Sectional Study to Assess Their Application in the General Population. Have We Achieved Our Goal?

**DOI:** 10.3390/ijerph17113980

**Published:** 2020-06-04

**Authors:** Stefano Palermi, Anna Maria Sacco, Immacolata Belviso, Veronica Romano, Pietro Montesano, Bruno Corrado, Felice Sirico

**Affiliations:** 1 Department of Public Health, University of Naples Federico II, 80131 Naples, Italy; stefano.palermi@unina.it (S.P.); annamaria.sacco@unina.it (A.M.S.); immacolata.belviso@unina.it (I.B.); veronica.romano@unina.it (V.R.); bruno.corrado@unina.it (B.C.); 2 Department of Motor Sciences and Wellness, University of Naples Parthenope, 80133 Naples, Italy; pieromontesano@libero.it

**Keywords:** guidelines, physical activity, sport, health, prevention

## Abstract

National and international healthcare organizations propose guidelines for physical activity worldwide, defining its characteristics. These guidelines’ practical applications are difficult to estimate, since they are not fully followed. The aim of the present cross-sectional observational study was to assess awareness about guidelines for physical activity and to evaluate their practical applications in a sample of the Italian population. In total, 310 participants completed an online survey (mean age 29.10 ± 4.44), assessing the habits, beliefs and health effects of physical activity. In total, 39.35% of respondents were inactive. In total, 6.91% of active respondents did not perform a warm-up phase at the beginning of each training session and 77.14% did not check their own heart rate during the training session. Approximately half of respondents reported erroneous beliefs about the type, frequency and volume of physical activity, compared to data proposed by the guidelines. The preventive effect of physical activity was clearly perceived for cardiovascular diseases, diabetes, metabolic syndrome and depression. Several subjects misinterpreted the preventive role of physical activity in colon and breast cancers, and in femur and vertebral fractures. Habits and beliefs about physical activity in the general population are far from the guidelines and recommendations. Therefore, it is necessary to strengthen the conscious practice of physical activity further.

## 1. Introduction

Physical inactivity is a worldwide problem [1] causing 3.2 million deaths per year globally, reaching fourth place in the list of risk factors [2]. Theoretically, complete “eradication” of physical inactivity would remove between 6% to 10% of the major non-communicable diseases [3].

Reducing physical inactivity is a main target of several international public health initiatives.

The “Global Action Plan for Physical Activity 2018–2030”, produced by the World Health Organization (“More active People for a Healthier World”), points to a 15% relative reduction in the global prevalence of physical inactivity in adults and in adolescents by 2030 [4].

Physical activity (PA), defined as any bodily movement produced by skeletal muscles that requires energy expenditure [5], is encouraged in the general population through the definition of new settings (i.e., schools, workplace, local communities), the higher awareness of health professionals in stimulating participation among health subjects and patients and through social and media advertisements to spread benefits related to PA.

The preventive [6] and therapeutic [7] role of PA [8] has been clearly defined under its metabolic, molecular, endocrinological, physiological and psychologic aspects [9,10]. PA has been considered as able to improve health, to avoid or to delay the onset of some non-communicable diseases and to improve well-being and quality of life [11]. These advantages have been proven across the entire human lifespan, from childhood [9] to old age [12,13]

The positive effects of PA have been proven in some subsets of patients with specific diseases (i.e., diabetes, hypertension, hypercholesterolemia and in different forms of disability [13,14,15]), gaining a first-line role in several therapeutic algorithms.

Indications and contraindications of PA have become a controversial discussion point among physicians and other health professionals [3,16]. Some research areas have contributed to defining risk factors related to PA and to implement algorithms and pre-participation screening strategies to avoid the harmful effects of PA in high-risk patients [17,18,19]. On the other hand, most national and international health organizations have proposed guidelines for PA, establishing the specific thresholds of type, duration, frequency and volume necessary to have positive effects on health [5].

Consequently, national organizations have proposed personal position statements about PA prescription protocols, like the UK [20], Australia [21] and the USA [22].

In Italy, although the Ministry of Health had produced national position statements encouraging participation in PA in the general population, nearly 40% of the adult population are still inactive [23], and nearly 30% of subjects are only partially active, performing an amount of PA below the national and international recommendations [23,24,25].

The reduction of these percentages of inactive or partially active subjects is a target of the Italian Government and the Ministry of Health and some activities are included in the “Essential Levels of Assistance”, a set of health activities guaranteed by the Italian National Health System. In their last publication (2017), actions to promote PA in specific settings like schools, the workplace and local communities and motivational counselling by trained health professionals have been included and defined as “essential” to promote health in the entire population. [26]

It is probable that a “gap” exists between theoretical evidence summarized in guidelines for PA and its dissemination in the general population, thus failing to reach a conscious and rational application. Some subjects report that they have no time to spend on this type of activity, so they perform an insufficient level of PA [27]. On the other hand, some active subjects assume that “more PA always means more benefits” determining inappropriate overuse stress on cardiovascular, musculoskeletal and other systems [8].

Moreover, beliefs about the positive effects of PA on the prevention of several diseases could be less perceived among active and inactive subjects, although these are clearly defined in the scientific literature supporting the guidelines’ recommendations [28].

Several studies aimed to investigate the presence and the entity of this “gap” between recommendations and practice in different geographical areas [29,30,31]. Strategies to define this problem and to speculate about reasons behind it are encouraged.

Therefore, the aim of the present study was to investigate habits and beliefs about PA in a cohort of Italian adult subjects. Habits about practiced PA and beliefs about potential positive effects of PA and about the amount of PA necessary to achieve relevant effects on health have been collected and discussed considering the WHO guidelines for PA adopted in Italy.

## 2. Materials and Methods

### 2.1. Participants

A cross-sectional observational study was carried out over a 1-month period (September 2019). For the scope of this study, a specific online survey was designed and edited in the participants’ native language (Italian) and was hosted on an online service for the study period (See Supplementary Tables S1 and S2). For the online survey, the authors used the Google Forms tool. Collected surveys were extracted in Microsoft Excel data sheets and formally adapted for import into STATA software v.12 (StataCorp. 2011, Stata Statistical Software: Release 12. StataCorp. LP, College Station, TX, USA). At the end of the study period, it was not possible to use the system of data collection again. The concept included in the survey was designed according to the World Health Organization guidelines for Physical Activity for Healthy Adults (aged 18–64) [5]. These guidelines have been adopted in Italy by the Minister of Health and are promoted in the general population. Italian national data about PA are organized and collected by the WHO according to these guidelines [32].

These guidelines are organized according to the age ranges considered: 5–17 years old, 18–64 years old and 65 years old and above.

For the purposes of the present study, guidelines for the 18–64-year-old group have been considered. According to the guidelines, the type of physical activity should take many different forms such as aerobic, strength, flexibility and balance to be effective.

In this age group, at least 150 min of moderate-intensity aerobic physical activity throughout the week (or at least 75 min of vigorous-intensity aerobic physical activity throughout the week or an equivalent combination of moderate- and vigorous-intensity activity) are suggested. Moreover, aerobic activity should be performed in bouts of at least 10 min duration and muscle-strengthening activities should be done by involving major muscle groups on 2 or more days a week.

The survey was completely anonymous, and participation was free and voluntary. The questionnaire was disseminated by sending a specific link to reach its online version through mail or social contacts, adopting a “snowball sampling” methodology. Although this methodology is limited by several sources of bias, the aim of the study was a cross-sectional picture of the perception and application of guidelines for PA in the general population and the random inclusion of subjects is guaranteed. No gender restriction was applied. If participants declared an age over or above 18–64 years at the beginning of the questionnaire, the survey was stopped automatically, and the attendee was not permitted to continue. No restriction on PA was applied and both inactive and active subjects were included.

After the collection of gender and age information, the survey was structured into 19 items organized in four main domains: habits about practiced PA, habits about performed warm-up, beliefs about PA recommendations, beliefs about relationship between PA and some diseases.

The first domain points to define subjects as active or inactive according to the level of PA performed during the last 12 months. Subjects were asked if they performed regular PA for at least three consecutive months during the last 12 months, to control for seasonal effects and recall bias.

No information about work activity was collected.

According to Italian national laws, subjects performing PA are differentiated into amateur/leisure athletes, non-agonistic athletes, agonistic athletes and professional athletes. These categories require different legal and medical management. Each active subject performing unstructured physical activity is defined as an amateur/leisure athlete (i.e., weekend runners). No medical certification is needed for this activity. Subjects performing structured PA, organized on behalf of National Federations of specific sports, are classified as non-agonistic, agonistic or professional, according to different criteria: age, gender and level of competition. Age and gender criteria are used to differentiate between non-agonistic and agonistic athletes for each sport. For both non-agonistic and agonistic athletes, a medical certification is required. Among agonistic athletes, some are classified as professional athletes, working for a sport society, with income, insurance and more consistent medical surveillance.

Since the Italian classification is not commonly used in other countries, from the perspective of the present study, non-agonistic, agonistic and professional athletes were considered together as “competitive athletes” in the data analysis.

Then, data about the type of PA performed, about the warm-up practice and the type and duration of exercises performed during warm-up were collected in subjects that stated that they practiced PA. Some specific questions related to PA were administrated during this section (i.e., Do you use any heart rate monitoring tool?).

Physical activity could include simple activity (i.e., walking) and no specific indication about warm-up and heart rate monitoring is directly defined by guidelines. Nevertheless, the authors included this section to define the practical application of methods to reduce the probability of PA-related injuries and methods in order to monitor the intensity of performed PA among active subjects engaged in structured programs of PA [5]. The participants who stated that they were inactive in the previous section of the survey skipped this section. Nevertheless, among inactive participants, the main reason to avoid PA was investigated (i.e., “Why do you not play sport?”). The third domain investigated beliefs about PA recommendation both in inactive and active respondents. In particular, the type of exercise (aerobic, strengthening and flexibility) that should be included as part of the PA section was questioned. Even beliefs about the amount of necessary PA per week were collected. The last section of the survey included questions aimed to investigate personal beliefs about the relationship between the effects of PA and some common diseases (i.e., “In your opinion, what is the role of Physical Activity against cardiovascular disease? Harmful, preventive, no effects, I don’t know”).

### 2.2. Data Collection and Analysis

According to the electronic design of the survey, only when respondents answered all questions was it possible to generate and send a report that was recordable in a general database. At the end of the study period, the complete database was extracted and analyzed using STATA software v.12 (StataCorp. 2011, Stata Statistical Software: Release 12. StataCorp. LP, Collage Station, TX, USA). Continuous variables were summarized and reported as the mean and standard deviation. Frequencies were reported as percentages. Only data from the second domain were analyzed in active respondents separately, while data about motivations for not performing PA were analyzed among inactive respondents. All the other domains were analyzed considering together data from inactive and active respondents. Indeed, the aim of the present study was to evaluate the awareness and the practical application of the guidelines for PA in a sample of the general population, including both inactive and active subjects. The specific perceptions of these subgroups were out of the scope of the present paper and further sub-analyses were not performed.

## 3. Results

The survey was completed by 310 participants: 158 (50.97%) females and 152 (49.03%) males. The mean age of subjects was 29.10 ± 4.44 years (range: 19–63). In total, 188 (60.65%) participants (88 females and 100 males, mean age 29.04 ± 4.98) practiced PA in the last 12 months (at least 3 months consecutively), regularly. In total, 122 (39.35%) participants did not perform PA in the last 12 months (70 females and 52 males, mean age 29.18 ± 4.38).

### 3.1. Active Subjects

Among active respondents, 80 (42.55%) were competitive athletes, while 108 (57.45%) were amateur athletes and practiced PA in leisure time. Several types of PA were performed by active respondents. Their frequencies and percentages are reported in Table 1.

The number of sessions of PA per week and duration of sessions (in minutes) are reported in Table 2. The average number of sessions of PA per week was 2.97 (median: three sessions of PA per week). Most of the active respondents performed sessions of PA between 60 and 90 min.

Regarding warm-up habits, 146 (77.67%) active respondents stated that they practiced a regular and structured warm-up before each training session. In total, 17 subjects (9.04%) declared that they carried out a warm-up before less than half of their training sessions, while 12 (6.38%) did so before over half their sessions. On the other hand, 13 subjects (6.91%) declared that they did not perform a warm-up before training sessions.

The warm-up phase had different durations among respondents, ranging from 5 min in 39 active subjects (22.28%) to 30 min in five active subjects (2.86%). Most active subjects performed a warm-up phase lasting 10 min (60 subjects; 34.23%).

Data about heart rate monitoring during PA were recorded. In total, 145 subjects (77.14%) did not check their hear rate during training sessions or warm-ups; 21 (11.17%) used a heart rate monitor, 12 (6.38%) measured their heart rate manually during the session, eight (4.25%) used a smartphone or smartwatch application for this purpose and two (1.06%) verified their heart rate with a gym machine directly.

### 3.2. Inactive Subjects

In total, 122 respondents, 39.35% of the whole sample declared that they had not practiced PA during the last 12 months and were classified as inactive. Among them, the reasons why PA was not practiced were investigated. In total, 77 (63.11%) declared to have not enough time to practice PA, 24 (19.67%) reported to have not sufficient motivation to practice PA, eight (6.57%) subjects had retired after an injury, six (4.91%) declared that they do not like PA, five (4.10%) thought it was too expensive, one (0.82%) was pregnant during the last year and one (0.82%) had started to practice PA but for less than 3 months and irregularly.

### 3.3. Beliefs about Characteristics of Physical Activity

Beliefs about characteristics of PA were investigated among active and inactive subjects. The proposed answers were tailored according to recommendations reported by WHO guidelines for PA in adults aged 18–64 [5]. For this population, at least 150 min per week of moderate-intensity aerobic activity (or 75 min of vigorous-intensity aerobic activity per week) are recommended, meaning at least 30 min daily during 5 days of the week.

In total, 190 (61.47%) subjects answered this question correctly. In total, 76 (24.60%) subjects considered 60 min of moderate-intensity aerobic activity daily for 5 days of the week necessary to obtain positive health effects. Only three (0.82%) subjects believed that at least 120 min of moderate-intensity aerobic activity daily (for 5 days of the week) was needed to obtain positive effects related to PA. In total, 41 (13.11%) subjects stated that there is not a specific minimum duration for PA.

According to WHO guidelines for PA, training sessions should include aerobic exercises, in bouts of at least 10 min in duration. Moreover, muscle-strengthening exercises involving large muscle groups should be performed 2 days a week and some exercises for flexibility should be carried out during the cooling down phase at the end of each session. According to the survey, 150 subjects (48.36%) considered it necessary to perform aerobic exercises only (i.e., running, cycling, swimming). Five subjects (1.63%) included muscle-strengthening exercises in PA sessions exclusively and 13 subjects (4.09%) included flexibility activities (i.e., stretching) exclusively. Only 142 subjects (45.90%) identified the combination of all three types of exercises (aerobic, muscle-strengthening and flexibility) correctly.

### 3.4. Beliefs about Relationship between Physical Activity and Some Diseases

Previous evidence has proven the beneficial and preventive effects of PA in some common diseases. These concepts are reported in WHO guidelines for PA, in which the preventive role of PA was clearly highlighted for cardiovascular diseases, diabetes, metabolic syndrome, colon cancer, breast cancer, femur fractures, vertebral fractures and depression, based on the available scientific literature. The last domain of the survey investigated the perceived relationship between the effect of PA and these diseases: “In your opinion, what is the role of Physical Activity against these diseases?”. Each subject could choose “Harmful”, “Preventive”, “No Effect” or “I don’t know”. Data are reported in Table 3.

## 4. Discussion

The results of the present study allow us to discuss several statements about habits related to the practical application of guidelines for PA in the general population and about beliefs regarding its effects on health. Almost 40% of respondents were considered inactive, not performing regular PA. Unfortunately, this data is even more alarming, as subjects included in the sample of the present study were young adults, with a mean age of 29.18 years. This percentage is almost constant, and it is in line with data from previous studies [17,33], which highlight that a large portion of the population performs levels of PA below those recommended. Moreover, evidence suggests that regular PA among young people is declining [34] and being physical inactive during young adulthood can determine severe health effects later in life [35].

In Italy, several national initiatives are attempting to improve this aspect and to “develop a culture of an active lifestyle for the entire population and especially young people, socially disadvantaged groups and frail older adults” [32].

To this scope, the level of physical activity in Italy is monitored through a national program, defined as “Progressi delle Aziende Sanitarie per la Salute in Italia (PASSI)” [23] in the age group 18–69, and other specific programs exist for different age ranges of subjects.

According to this program, “active subjects” are subjects meeting the WHO PA guidelines recommendations or performing a heavy work activity. “Partially active subjects” are subjects performing a heavy work activity without reaching the level of PA recommended by WHO in leisure time, “sedentary subjects” are subjects with a sedentary work activity, that do not reach the level of PA suggested by WHO guidelines. The most recent data (2015–2018) of this report indicate that 31.4% of the population is “active”, 34.1% is “partially active” and 34.5% is “sedentary”. Although the data of the present study do not allow differentiation between “active” and “partially active” based on work activity, our data about the percentage of inactive subjects are similar to the data reported in the PASSI program for “sedentary” subjects. Some “partially active” and “sedentary” subjects (one out of two and one out of five, respectively) have misperceptions of their PA status, perceiving their level of PA as sufficient. Interestingly, the PASSI report highlights a low degree of medical attention being paid to this problem. Indeed, a rather low percentage of subjects classified as partially active or sedentary, even suffering from other clinical conditions or being overweight, have received sufficient support and advice by medical doctors or other healthcare professionals (less than 40% of overweight subjects and less than 45% of subjects with other chronic diseases). Nevertheless, a percentage of the population is still inactive worldwide [36], in Italy [23], and according to the data from the present study.

Reasons to be inactive are different and difficult to assess sometimes [37]. The main reason for participants not performing PA in the present study was related to the amount of time required to perform it (almost 60% of inactive subjects stated this opinion). The PA is often perceived as a heavy “obligation” that requires a lot of time and effort [38,39]. It is possible that the general opinion of PA is associated to professional sport, forcing subjects to think they have to dedicate several hours daily to exercise in order to obtain significant results [40].

This concept is not sustained nor promoted by guidelines for PA. Scientific evidence supports the concept that at least 30 min daily PA for 5 days/week is enough to determine relevant positive effects on health [5]. This recommendation about the volume of PA is perceived only by almost 60% of respondents correctly. On the other hand, this data suggests that, for 40% of respondents, it is necessary to dedicate more time to PA to reach some clinical advantages. Almost 20% of inactive subjects declared that they were not motivated to perform PA. A lack of time [27] and poor motivation [41] are well-known barriers to PA practice. Both of these negative factors could be related to a poor awareness about guidelines for PA in the general population. Indeed, little efforts have been taken to disseminate these guidelines [29], and some benefits of PA for health are not known, although they are scientifically proven.

This problem has already been investigated in other geographical areas. For example, Kay et al. [30] found that only 36% of the national survey’s respondents were aware of PA recommendations in the USA. Similarly, recommendations about PA were correctly recalled by only 15% of respondents in the UK [31]. Moreover, only 4.4% of Chinese college students had correct knowledge of PA recommendations [29]. To our best knowledge, no similar previous studies have been conducted among Italian adults. However, data from the present study are in line with those from studies including subjects from other countries.

It is interesting to consider that a large part of the population is engaged in amateur organized and structured activities (i.e., gym activity, individual sports, running, cycling and others) with a planned and scheduled training session [42,43].

In Italy and in other countries, no medical examinations are required for these subjects. They are strongly encouraged to practice PA, but the type of PA performed is mainly based on personal preferences or on the advice of non-health professionals.

In this subset of subjects, the correct perception of guidelines is a public health need. Disseminating valid advice about the type, frequency, volume, indications and contraindications of PA is important and relevant to the medical field. While competitive athletes have planned surveillance of their activities, amateur athletes have different sources of knowledge about the characteristics of PA. This might determine the adoption of habits not useful to reach adequate level of PA (immoderate use of food supplements, drug abuse to improve performance, overtraining programs increasing the risk of injuries) [33,44,45,46].

In some settings, like the workplace of big and medium factories, some programs have been implemented to encourage these lifestyles and to spread the effectiveness of PA as a preventative approach [47].

From this perspective, it is necessary to encourage the practice of preventive behavior able to reduce the main injuries inherited in relation to structured PA activities (i.e., musculoskeletal injuries) [48,49,50]. In this regard, several protocols for warm-ups have been proposed [51,52] and their investigation has been partially included in the present survey.

Although a large amount of evidence in the literature supports the health and preventive benefits of a well-conducted warm-up [53], it is not always performed regularly. Indeed, almost 7% of respondents do not practice a structured warm-up before training sessions and 9% of subjects declared that they did this less than half the time. It is possible that more strategies aimed to spread the preventive utility of warm-ups as a method to avoid some sport injuries should be conducted, adopting specific and proven protocols [52]. The type of exercises included in the warm-up routine, the adoption of specific forms of stretching and the exact timing of these activities to maximize their preventive role are still debated. Nevertheless, the inclusion of a warm-up protocol before a training session in amateur and professional athletes has shown positive outcomes in deterring injuries [52].

Nearly 73% of subjects do not check their heart rate during sessions of PA. The development of new and simple heart rate monitors has rapidly evolved during the past two decades [54] and they have become a widely used training aid for a variety of sport. Monitoring the training volume and athletes’ cardiovascular responses is crucial to make informed decisions about training and recovery prescriptions [55]. Often, the prescription of PA among adult subjects is made according to a percentage of the Maximal Theoretical Heart Rate (MTHR), calculated commonly as 220 minus the person’s age [56] (which should be reached and not surpassed during each session) and it is suggested to keep the heart rate within a specific range of MTHR (i.e., “practice activity maintaining your heart rate between 60–80% of MTHR”). It is almost impossible to control this variable without the aid of a heart rate monitoring methodology. Although the guidelines do not clearly state the use of this methodology, they include heart rate monitoring among the methods to assess the intensity of PA. Monitoring exercise intensity through heart rate assessment is cheap, easy and can be used in most situations. In addition, this method could be useful in the prevention and detection of overtraining [54].

Furthermore, the type of PA performed was also investigated. Physical activity guidelines recommend including other exercises beyond aerobic activities, such as muscle-strengthening exercises and flexibility exercises. In particular, muscle-strengthening exercises involving large muscle groups should be performed at least twice per week, while flexibility exercises should be part of a cooling down phase at the end of each session of PA. Almost half of the subjects in our sample were focused on aerobic exercises only, leaving out other forms of exercise [5]. This concept is relevant because aerobic exercises can promote several positive effects, but muscle-strengthening exercises are a proven method to limit sarcopenia during late adulthood [57]. Moreover, flexibility exercises are necessary to avoid muscle injuries and overuse lesions [58]. Therefore, it is important to reinforce the concept of a multimodal session of PA in recommendations for the general population.

The last section of the survey investigated beliefs about the relationship between PA and some common diseases. Over 90% of subjects declared that PA has a preventive effect on cardiovascular diseases and depression. Compared to less active adult men and women, individuals who are more active have lower rates of all-cause mortality, coronary heart disease and high blood pressure. Preventive strategies, based on correct and active lifestyles, represent one of the most powerful resources to reduce the burden of cardiovascular diseases [47]. Similarly, almost 80% of respondents sustained the preventive effect of PA on diabetes and metabolic syndrome. Conversely, interesting results come from the analysis of the association between PA and fractures (femoral and vertebral fractures) and between PA and cancer (colon and breast cancers). The effects of PA on these diseases are less perceived in the general population. Nevertheless, a large amount of evidence includes PA as a crucial factor in the prevention algorithm of osteoporosis [59]. Similarly, a plethora of evidence shows that regular PA decreases the risk of colon and breast cancers [60]. Unfortunately, almost half of participants only perceived the preventive effect of PA about these diseases correctly. A large percentage of subjects were not able to make a definite judgment about the relationship between PA and this subset of diseases, and some even perceived PA as a risk factor for them.

The analysis of these data should be useful to plan future public health campaigns aimed to highlight the preventive role of PA on osteoporosis and cancers. Indeed, this questionnaire demonstrates that opinions about this topic in the general population are significantly far from concepts suggested by guidelines for PA.

Admittedly, this study suffers for some limitations. First, although 310 subjects were included in the analysis, this sample is limited to obtain a detailed “picture” of the adult population aged 18–64. Nevertheless, data analysis produced results in agreement with previous similar findings in other geographical areas and could be useful to design and conduct future larger studies in the Italian population to better investigate specific issues about recommendations for PA. The method that was adopted to distribute the survey relied on a “snowball” methodology. Although this methodology was limited by several sources of bias, it was acceptable according to the specific aim of the present study, in the authors’ opinion. Indeed, the main bias of this methodology is that each subject was invited to contribute to the dissemination of the survey. Obviously, this dissemination relies on subjects’ characteristics and active subjects are more prone to include other active subjects (i.e., their teammates), while inactive subjects do the same. Nevertheless, the aim of the study was to paint a picture of the perception and application of guidelines for PA in the general population. The structure of the survey did not include a specific session to investigate comorbidity specifically. Some subjects in the present sample could have been diabetic or suffered from hypertension or other cardiovascular diseases. These data have been not collected. Nevertheless, this paper aimed to investigate habits and beliefs about the awareness and application of guidelines for PA. Although some patients require specific guidelines for the prescription of PA (i.e., patients affected by diabetes), perspectives about PA are based on subjects’ opinions, analyzed through the survey.

Furthermore, the method adopted to define a subject as active or inactive has some limitations. Being “active” is not strictly related to the concept of practicing a structured program of exercise. Walking regularly and adopting a more active lifestyle (using stairs, parking far away from destinations, avoiding the overuse of cars, and other activities) could reach the thresholds of PA defined by the guidelines.

In this assessment, it is important to also consider work activity. Different work activities could be related to different levels of PA.

At the same time, some subjects could be erroneously defined as active, because they reach some thresholds defined by the guidelines but avoid some forms of PA (i.e., muscle-strengthening exercises) and thus overestimate their level of PA.

These aspects are difficult to quantify though a survey and could be underestimated by our data, by improperly increasing the percentage of subjects defined as “active” or “inactive”.

For these reasons, we have decided to avoid a subgroup analysis based on the distinction between active and inactive subjects.

Finally, it is necessary to underline that guidelines should not be considered as strict rules in a healthcare system, but as a “collection” of recommendations based on scientific evidence. Indeed, guidelines are recommendations regarding clinical behavior, which are made through a systematic revision process of the medical literature and specialists’ opinions. Their aim is to help doctors and patients to make the best decisions about treatment for a particular condition, by choosing the most appropriate strategies in specific clinical situations [61]. Therefore, to follow guidelines for PA and to plan strategies for their distribution in the general population should be considered a reasonable and effective approach to improve health.

Beyond these limitations, the results of the present study underline that guidelines for PA proposed by relevant international organizations like the WHO do not have a “capillary” distribution across the general population. Some habits and beliefs about PA are distorted and incorrect. This could determine a reduction in participation in PA programs among inactive subjects. On the other hand, poor awareness about guidelines for PA could determine overuse injuries and incorrect habits among active subjects. The perception of the preventive effects of PA on some diseases, such as fractures and cancers, are not fully perceived by the general population, determining their misperceptions and incorrect attitudes about this relevant topic.

Although, in Italy, PA levels are monitored continuously through national surveillance systems promoted by the Ministry of Health, such as PASSI [23] and others (i.e., the “Okkio alla salute” program, a Child Obesity Surveillance Initiative collecting data on children aged 8–9 years), the results of the present study suggest that further initiatives are necessary to implement a general consciousness about recommendations for PA.

## 5. Conclusions

Almost 40% of the study population was considered inactive. A lack of time and poor motivation to perform PA were the main causes of physical inactivity. Among active subjects, warm-up protocols were not practiced by 7% and were practiced irregularly by 9% of subjects. Heart rate monitors were poorly used during PA sessions. The volume and type of PA recommended for adults aged 18–64 by WHO guidelines for PA were ignored by a large percentage of subjects. The preventive effects of PA in relation to cardiovascular diseases, diabetes, metabolic syndrome and depression were perceived by the general population correctly. Nevertheless, although supported by scientific evidence and recommended by the guidelines, the preventive effects of PA in relation to femoral and vertebral fractures and colon and breast cancers were still underestimated by participants. These results suggest that an improvement is required to ensure the extensive dissemination of guidelines for PA across the general population, increasing the awareness about the type, volume and frequency of PA able to have positive effects on health. Furthermore, our results could be useful in the planning of strategies to improve people’s perception of the preventive effects of PA in relation to some common diseases, such as osteoporosis and cancers.

## Figures and Tables

**Table 1:** Types and frequencies of physical activity (PA) performed by active respondents.

Physical Activity	N	%
Gym activity *	84	44.68%
Football	24	12.77%
Swimming	22	11.70%
Running	19	10.11%
Weight Lifting	11	5.85%
Football Referee	9	4.79%
Martial Arts	4	2.13%
Volleyball	3	1.60%
Rugby	2	1.06%
Dance	2	1.06%
Diving	1	0.53%
Water Aerobics	1	0.53%
Rowing	1	0.53%
Cycling	1	0.53%
Equitation	1	0.53%
Motorcycling	1	0.53%
Figure Skating	1	0.53%
Boxing	1	0.53%
TOTAL	*n* = 188	100.00%

* Gym activity included different types of PA (fitness, aerobic, free body exercises, gymnastic, crossfit, athletic, fit box).

**Table 2:** Training sessions per week and single session durations (in minutes) among active respondents.

Training Sessions per Week	*n*	%
2	67	35.64%
3	82	43.62%
4	19	10.11%
5	18	9.57%
6	1	0.53%
7	1	0.53%
TOTAL	*n* = 188	100.00%
**Single Session Duration (min)**	***n***	**%**
45	40	21.28%
60	77	40.96%
90	58	30.85%
120	11	5.85%
more than 120	2	1.06%
TOTAL	*n* = 188	100.00%

**Table 3:** Perceived effects of PA on different diseases.

Diseases	Harmful	Preventive	No Effect	I Don’t Know
Cardiovascular Diseases	0.83%	95.04%	1.65%	2.48%
Diabetes	0.90%	81.08%	7.21%	10.81%
Metabolic Syndrome	0.78%	79.12%	5.45%	15.45%
Colon cancer	5.83%	47.57%	21.36%	25.24%
Breast cancer	5.88%	38.24%	26.47%	29.41%
Femur fracture	15.69%	55.88%	12.75%	15.69%
Vertebral fracture	16.35%	56.73%	9.62%	17.31%
Depression	0%	93.16%	4.27%	2.56%

## Supplementary Materials

The following are available online at https://www.mdpi.com/1660-4601/17/11/3980/s1, Table S1: Survey (Italian version), Table S2: Survey (English version).
