# Supplementary material for: Guidelines for Physical Activity—A Cross-Sectional Study to Assess Their Application in the General Population. Have We Achieved Our Goal?
Source: Int J Environ Res Public Health. 2020 Jun 4;17(11):3980. doi: 10.3390/ijerph17113980 (PMC7313455; doi:10.3390/ijerph17113980)
Supplement: Supplementary file 1 [file ijerph-17-03980-s001.zip › Table S1 - Survey Italian version.docx]

**Table S1.** Survey (Italian version)

|  | **Domanda** | **Risposta** |
| --- | --- | --- |
| **GENERALI** | | |
|  | Sesso | Maschio |
|  |  | Femmina |
|  | Età |  |
| **DOMINIO 1 (tutti i soggetti)** | | |
| 1 | Hai praticato sport negli ultimi 12 mesi, almeno 3 mesi consecutivamente? | Si (vai alla 2) |
|  |  | No (vai alla 2bis) |
| **DOMINIO 2 (soggetti attivi)** | | |
| 2 | Che sport pratichi? |  |
| 3 | A che livello? | Amatoriale |
|  |  | Non Agonistico* |
|  |  | Agonistico* |
| 4 | Quanti allenamenti a settimana fai? | 1 |
|  |  | 2 |
|  |  | 3 |
|  |  | 4 |
|  |  | 5 |
|  |  | 6 |
|  |  | 7 |
| 5 | Quanto dura in media un tuo allenamento (min)? | 30 |
|  |  | 45 |
|  |  | 60 |
|  |  | 90 |
|  |  | 120 |
|  |  | > 120 |
| 6 | Fai una fase di riscaldamento prima di iniziare l’allenamento? | Si |
|  |  | No |
|  |  | Meno della metà delle volte |
|  |  | Più della metà delle volte |
| 7 | Quanto dura la tua fase di riscaldamento (min)? | 5 |
|  |  | 10 |
|  |  | 15 |
|  |  | 20 |
|  |  | 25 |
|  |  | 30 |
|  |  | > 30 |
| 8 | Utilizzi qualche metodo per monitorare la frequenza cardiaca durante il riscaldamento o l’allenamento? | Cardiofrequenzimetro |
|  |  | Misurazione manuale |
|  |  | Applicazioni per smartphone/smartwatch |
|  |  | Altro (specificare) |
| **DOMINIO 2 (soggetti inattivi)** | | |
| 2bis | Perché non fai sport? (vai alla 9) | Mancanza di tempo |
|  |  | Mancanza di motivazione |
|  |  | Ho smesso dopo un infortunio |
|  |  | Non mi piace |
|  |  | Altro (specificare) |
| **DOMINIO 3 (tutti i soggetti)** | | |
| 9 | Secondo te, quale è il tempo minimo necessario da spendere in attività fisica a moderata intensità per ottenere benefici in termini di salute? NB: Per moderata intensità si intende uno sforzo pari a 5-6 (in scala da 0 a 10 dove 0 corrisponde al riposo e 10 al massimo sforzo possibile) | 30 min al giorno per 5 giorni a settimana o 50 min al giorno per 3 giorni a settimana (150 min totali a settimana) |
|  |  | 60 min al giorno per 5 giorni a settimana (300 min a settimana) |
|  |  | 120 min al giorno per 5 giorni a settimana (600 min a settimana) |
|  |  | Non esiste un tempo specifico |
| 10 | Secondo te, un programma di esercizio fisico in persone tra i 18 e i 64 anni, che possa dare dei benefici in termini di salute, dovrebbe comprendere | Esercizi aerobici (correre, camminare velocemente, andare in bicicletta, nuotare) |
|  |  | Esercizi per forza muscolare (sollevamento pesi, esercizi a corpo libero, esercizi con macchine) |
|  |  | Stretching per migliorare la flessibilità e l’elasticità muscolare |
|  |  | Esercizi aerobici, esercizi per la forza muscolare e stretching |
| 11 | Le attività aerobiche (correre, camminare velocemente, nuotare, andare in bicicletta) dovrebbero essere svolte continuativamente per almeno | 10 min |
|  |  | 20 min |
|  |  | 30 min |
|  |  | 60 min |
|  |  | Fino all’esaurimento muscolare |
| **DOMINIO 4 (tutti i soggetti)** | | |
| 12 | Secondo te, l’attività fisica regolare che effetto ha sulle seguenti malattie? |  |
|  | - malattie cardiovascolari | Dannoso |
|  |  | Preventivo |
|  |  | Nessun effetto |
|  |  | Non saprei |
| 13 | Secondo te, l’attività fisica regolare che effetto ha sulle seguenti malattie? |  |
|  | - diabete | Dannoso |
|  |  | Preventivo |
|  |  | Nessun effetto |
|  |  | Non saprei |
| 14 | Secondo te, l’attività fisica regolare che effetto ha sulle seguenti malattie? |  |
|  | - sindrome metabolica | Dannoso |
|  |  | Preventivo |
|  |  | Nessun effetto |
|  |  | Non saprei |
| 15 | Secondo te, l’attività fisica regolare che effetto ha sulle seguenti malattie? |  |
|  | - tumore al colon | Dannoso |
|  |  | Preventivo |
|  |  | Nessun effetto |
|  |  | Non saprei |
| 16 | Secondo te, l’attività fisica regolare che effetto ha sulle seguenti malattie? |  |
|  | - tumore alla mammella | Dannoso |
|  |  | Preventivo |
|  |  | Nessun effetto |
|  |  | Non saprei |
| 17 | Secondo te, l’attività fisica regolare che effetto ha sulle seguenti malattie? |  |
|  | - frattura del femore | Dannoso |
|  |  | Preventivo |
|  |  | Nessun effetto |
|  |  | Non saprei |
| 18 | Secondo te, l’attività fisica regolare che effetto ha sulle seguenti malattie? |  |
|  | - fratture vertebrali | Dannoso |
|  |  | Preventivo |
|  |  | Nessun effetto |
|  |  | Non saprei |
| 19 | Secondo te, l’attività fisica regolare che effetto ha sulle seguenti malattie? |  |
|  | - depressione | Dannoso |
|  |  | Preventivo |
|  |  | Nessun effetto |
|  |  | Non saprei |
