# Supplementary material for: Guidelines for Physical Activity—A Cross-Sectional Study to Assess Their Application in the General Population. Have We Achieved Our Goal?
Source: Int J Environ Res Public Health. 2020 Jun 4;17(11):3980. doi: 10.3390/ijerph17113980 (PMC7313455; doi:10.3390/ijerph17113980)
Supplement: Supplementary file 1 [file ijerph-17-03980-s001.zip › Table S2 - Survey English version.docx]

**Table S2.** Survey (English version)

|  | **Question** | **Answer** |
| --- | --- | --- |
| **GENERAL** | | |
|  | Sex | Male |
|  |  | Female |
|  | Age |  |
| **DOMAIN 1 (all subjects)** | | |
| 1 | Did you perform regular physical activity during the last 12 months (for at least 3 months consecutively)? | Yes (go to 2) |
|  |  | No (go to 2bis) |
| **DOMAIN 2 (active subjects)** | | |
| 2 | Which kind of physical activity do you perform? |  |
| 3 | Which level of physical activity do you perform? | Amateur |
|  |  | Non Agonistic^1^ |
|  |  | Agonistic^1^ |
| 4 | How many times a week do you train? | 1 |
|  |  | 2 |
|  |  | 3 |
|  |  | 4 |
|  |  | 5 |
|  |  | 6 |
|  |  | 7 |
| 5 | How long does your training last (min)? | 30 |
|  |  | 45 |
|  |  | 60 |
|  |  | 90 |
|  |  | 120 |
|  |  | > 120 |
| 6 | Do you have a warm-up phase before training? | Yes |
|  |  | No |
|  |  | Less than half the time |
|  |  | More than half the time |
| 7 | How long does your warm-up phase last (min)? | 5 |
|  |  | 10 |
|  |  | 15 |
|  |  | 20 |
|  |  | 25 |
|  |  | 30 |
|  |  | > 30 |
| 8 | Do you use any heart rate monitoring tool? | Heart rate monitor |
|  |  | Manual measurement |
|  |  | Smartphone/smartwatch application |
|  |  | Other (please, specify) |
| **DOMAIN 2 (inactive subjects)** | | |
| 2bis | Why did you not perform regular physical activity? (go to 9) | Lack of time |
|  |  | Lack of motivation |
|  |  | Retired after injury |
|  |  | I don’t like |
|  |  | Other (please, specify) |
| **DOMAIN 3 (all subjects)** | | |
| 9 | In your opinion, what is the minimum time needed to spend in moderate intensity physical activity to obtain health benefits? NB: a moderate intensity physical activity is a 5-6 points effort (in a 0-10 scale, where 0 is rest and 10 is the maximum effort possible) | 30 min daily for 5 days a week or 50 min daily for 3 days a week (150 min weekly overall) |
|  |  | 60 min daily for 5 days a week (300 min weekly overall) |
|  |  | 120 min daily for 5 days a week (600 min weekly overall) |
|  |  | There is no specific time |
| 10 | In your opinion, a healthy physical activity program for 18-64 aged people, should be made of | Aerobic activity (running, fast walking, bicycling, swimming) |
|  |  | Strengthening activity (weightlifting, free body exercises, gym machines exercises) |
|  |  | Stretching activity, to improve muscular flexibility |
|  |  | Aerobic activity, strengthening activity, stretching activity |
| 11 | Aerobic activity (running, fast walking, bicycling, swimming) should be consecutively played for at least | 10 min |
|  |  | 20 min |
|  |  | 30 min |
|  |  | 60 min |
|  |  | Until muscular exhaustion |
| **DOMAIN 4 (all subjects)** | | |
| 12 | In your opinion, what is the role of physical activity against these diseases? |  |
|  | - cardiovascular disease | Harmful |
|  |  | Preventive |
|  |  | No effects |
|  |  | I don’t know |
| 13 | In your opinion, what is the role of physical activity against these diseases? |  |
|  | - diabetes | Harmful |
|  |  | Preventive |
|  |  | No effects |
|  |  | I don’t know |
| 14 | In your opinion, what is the role of physical activity against these diseases? |  |
|  | - metabolic syndrome | Harmful |
|  |  | Preventive |
|  |  | No effects |
|  |  | I don’t know |
| 15 | In your opinion, what is the role of physical activity against these diseases? |  |
|  | - colon cancer | Harmful |
|  |  | Preventive |
|  |  | No effects |
|  |  | I don’t know |
| 16 | In your opinion, what is the role of physical activity against these diseases? |  |
|  | - breast cancer | Harmful |
|  |  | Preventive |
|  |  | No effects |
|  |  | I don’t know |
| 17 | In your opinion, what is the role of physical activity against these diseases? |  |
|  | - femur fracture | Harmful |
|  |  | Preventive |
|  |  | No effects |
|  |  | I don’t know |
| 18 | In your opinion, what is the role of physical activity against these diseases? |  |
|  | - vertebral fractures | Harmful |
|  |  | Preventive |
|  |  | No effects |
|  |  | I don’t know |
| 19 | In your opinion, what is the role of physical activity against these diseases? |  |
|  | - depression | Harmful |
|  |  | Preventive |
|  |  | No effects |
|  |  | I don’t know |

^1^ Distinction based on Italian national Laws. See the text for detailed explanation.
